# 2025 National Guidelines on the Mediterranean Diet: Executive Summary of a Joint Report by Italian Scientific Societies and the National Institute of Health Task Force on Clinical Practice Guidelines

**DOI:** 10.1093/nutrit/nuaf263

**Published:** 2026-01-05

**Authors:** Nicola Veronese, Vincenza Gianfredi, Massimo Volpe, Michela Zanetti, Graziano Onder, Marco Silano, Daniele Nucci, Luigi Fontana, Alessandro Laviano, Cornel Sieber, Stefania Maggi, Italian Mediterranean Guideline Group, Stefania Maggi, Massimo Volpe, Michela Zanetti, Graziano Onder, Nicola Veronese, Vincenza Gianfredi, Daniele Nucci, Marco Silano, Laura Rossi, Brunello Antonella, Caffarra Paolo, Cotroneo Antonio Maria, Ciccone Marco Matteo, Desideri Giovambattista, Galletti Ferruccio, Guberti Emilia, Lezo Antonella, Leonardi Francesco, Macchia Francesco, Maiorino Maria Ida, Medea Gerardo, Paolini Barbara, Rogoli Domenico, Scarpato Elena, Sciretti Massimiliano, Spagnuolo Maria Immacolata, Troiano Ersilia, Ungar Andrea, Roberto Volpe, Zuliani Giovanni, Alonzo Elena, Aversano Fiorella, Baldini Letizia, Barbarano Federico, Benussi Alberto, Burgio Marianna Ilarj, Buscemi Carola, Camastra Stefania, Cappiello Ilaria, Caprio Massimiliano, Caruso Irene, Casirati Amanda, Ceriani Elisa, Chernykh Anastasia, Chimienti Martina, Chiriacò Martina, Cicero Arrigo Francesco Giuseppe, Cilona Laura, Cintoni Marco, Ciriminna Stefano, Crudele Lucilla, D’Eusebio Chiara, Deledda Andrea, Di Lorenzo Francesco, Espa Lorenzo, Fadda Maurizio, Favaro Enrica, Fichera Martina, Ferrera Armando, Galluzzo Lucia, Giussani Cristina, Greatti Alessandro, Guindani Paola, Limongi Federica, Lisso Fabrizia, Mazza Elisa, Misotti Alessandro, Mollica Maria Pina, Morlando Alessia, Morsella Alisha, Muscogiuri Giovanna, Pala Barbara, Paolillo Stefania, Parretti Damiano, Patti Maria Cristina, Pira Costanza, Ragusa Francesco Saverio, Randazzo Cristiana, Romiti Giulio Francesco, Salatto Alessia, Schirò Piero, Sollai Sara, Testa Giuseppe Dario, Trestini Ilaria, Valoriani Filippo, Velluzzi Fernanda, Zimmitti Salvatore, Alisha Morsella, Federica Limongi, Francesco Saverio Ragusa, Luigi Fontana, Alessandro Laviano, Cornel Sieber

**Affiliations:** Saint Camillus International University of Health Sciences, Faculty of Medicine, 00161, Rome, Italy; Unità Locale Socio-Sanitaria 3 “Serenissima”, Primary Care Department, Venice, Italy; Department of Biomedical Sciences for Health, University of Milan, 20133, Milan, Italy; IRCCS San Raffaele Roma, Rome, Italy; Department of Clinical and Molecular Medicine Sapienza—Università di Roma, Italy; Department of Medicine, Surgery and Health Sciences, University of Trieste, Trieste, Italy; Department of Geriatrics, Università Cattolica del Sacro Cuore, 00168, Rome, Italy; Palliative Care Unit, Fondazione Policlinico Gemelli IRCCS, 00168, Rome, Italy; Department of Cardiovascular, Endocrine-Metabolic Diseases and Aging, Italian National Institute of Health, 00161, Rome, Italy; Struttura Semplice Dipartimentale Igiene Alimenti e Nutrizione, Dipartimento di Igiene e Prevenzione Sanitaria, Agenzia di Tutela della Salute (ATS) Brescia, 25124, Brescia, Italy; National PhD Program in One Health Approaches to Infectious Diseases and Life Science Research, Department of Public Health, Experimental and Forensic Medicine, University of Pavia, 27100, Pavia, Italy; Chales Perkins Centre, Faculty of Medicine and Health, The University of Sydney, 2006, Sydney, Australia; Department of Endocrinology, Royal Prince Alfred Hospital, 2050, Sydney, Australia; Department of Translational and Precision Medicine, Sapienza University of Rome, Rome, Italy; Director, Institute for Biomedicine of Aging (IBA), Friedrich-Alexander-Universität Erlangen-Nürnberg, Germany; CMO, County Hospital Winterthur, Switzerland; National Research Council (CNR), Aging Section, Padova, Italy

**Keywords:** mediterranean diet, guidelines, mortality, cardiovascular, oncology, metabolism, pregnancy, GRADE

## Abstract

**Context:**

Chronic non-communicable diseases (NCDs) are the leading global causes of morbidity, mortality, and escalating health care costs, driven largely by unhealthy lifestyles in an aging population.

**Objective:**

The Mediterranean Diet (MD), traditionally associated with healthy longevity, is increasingly recognized for its preventative and therapeutic benefits across many NCDs.

**Data sources:**

Using the NUTRIGRADE methodology, a multidisciplinary panel of clinical, methodological, and public health experts conducted a comprehensive systematic review across 9 health domains and one economic domain.

**Data extraction:**

Data were extracted from multiple databases without date restrictions, assessing outcomes such as disease incidence and prevalence, mortality rates of nutrition-related NCDs, quality of life indicators, and relevant biochemical markers. Evidence was critically appraised and synthesized to formulate evidence-based recommendations.

**Data analysis:**

A total of 84 evidence-based recommendations were developed. The MD was found to significantly reduce all-cause mortality in the general population and cardiovascular morbidity and mortality, particularly in high-risk individuals. High-quality evidence supports the protective role of the MD against myocardial infarction, stroke, atrial fibrillation, and peripheral arterial disease. In oncological evidence, MD adherence has been found to be linked to a lower incidence of several cancer types and improved survival outcomes. Additional benefits include reduced incidence and progression of cognitive decline, type 2 diabetes, metabolic syndrome, and obesity, and improved pregnancy outcomes. The strength of recommendations varied by outcome and target population, with many supported by robust evidence.

**Conclusions:**

The MD is a scientifically supported dietary intervention with broad preventive and therapeutic benefits. Its integration into clinical practice, public health strategies, and policy initiatives is strongly recommended to promote health and reduce the global health care burden.

## INTRODUCTION

Over the past 150 years, global life expectancy has increased dramatically, resulting in prolonged exposure to risk factors and a rising burden of noncommunicable diseases (NCDs).^1^^,^^2^ Unhealthy diets, sedentary lifestyle, smoking, and mental stress, now widespread, are key contributors to the onset and progression of age-associated chronic diseases, which are the leading cause of morbidity, mortality and functional decline, diminished quality of life, and increased dependency.^3^ These trends place significant pressure on health care systems, human resources, and economic sustainability, and highlight the need not only to extend life but to promote healthy aging by preventing multimorbidity and disability.^4^

In recent years, nutrition research has shifted focus from individual nutrients to overall dietary patterns, recognizing that food combinations can have synergistic or antagonistic effects on health.^5^ Among these, the Mediterranean diet (MD) has been extensively studied and is associated with reduced risk of multiple chronic disease and premature death.^6^ More than a dietary regimen, the MD reflects a holistic lifestyle rooted in cultural traditions, emphasizing minimally processed, seasonal, and biodiverse foods; conviviality; and physical activity.^7^ In recognition of its cultural significance, UNESCO (the United Nations Educational, Scientific and Cultural Organization) designated the MD as an intangible cultural heritage of humanity in 2010.^8^

Given the decline in adherence to the traditional MD—particularly in Mediterranean regions like Italy—and the growing burden of NCDs worldwide, there is a critical need to reinforce the relevance of the MD through scientifically grounded guidance.^9^ In this article we present a comprehensive set of updated, evidence-based clinical recommendations that synthesize the current state of knowledge on the role of the MD in the prevention and management of NCDs. By integrating the latest high-quality evidence, these guidelines aim to support health care professionals, policymakers, and the general public in promoting the MD as a central component of both primary and tertiary prevention strategies, with the ultimate goal of advancing healthy aging and reducing the global impact of chronic disease.

## METHODS

In this practical guideline we present clinical recommendations developed through a collaborative effort led by the Italian Society of Artificial Nutrition and Metabolism (Società Italiana di Nutrizione Artificiale e Metabolismo SINPE), Italian Society for Cardiovascular Prevention (Società Italiana per la prevenzione cardiovascolare [SIPREC]) and the Mediterranean Diet Foundation (Fondazione Dieta Mediterranea), with the support of the Italian National Institute of Health (Istituto Superiore di Sanità) and over 20 Italian scientific societies spanning both medical and non-medical disciplines and multiple stakeholders. A schematic overview of the project is presented in Figure 1. The original version of the guideline, in Italian, is published on the official Italian clinical guidelines website: https://www.iss.it/-/dieta-mediterranea. Following the Preferred Reporting Items for Systematic reviews and Meta-Analyses (PRISMA) 2020 framework,^10^ we conducted a comprehensive and systematic literature search on February 28, 2024. In this review we addressed 9 clinical questions framed using the PICO (Population, Intervention, Comparison, Outcome) format, along with an additional question focusing on economic and sustainability aspects of the MD.

**Figure 1.**
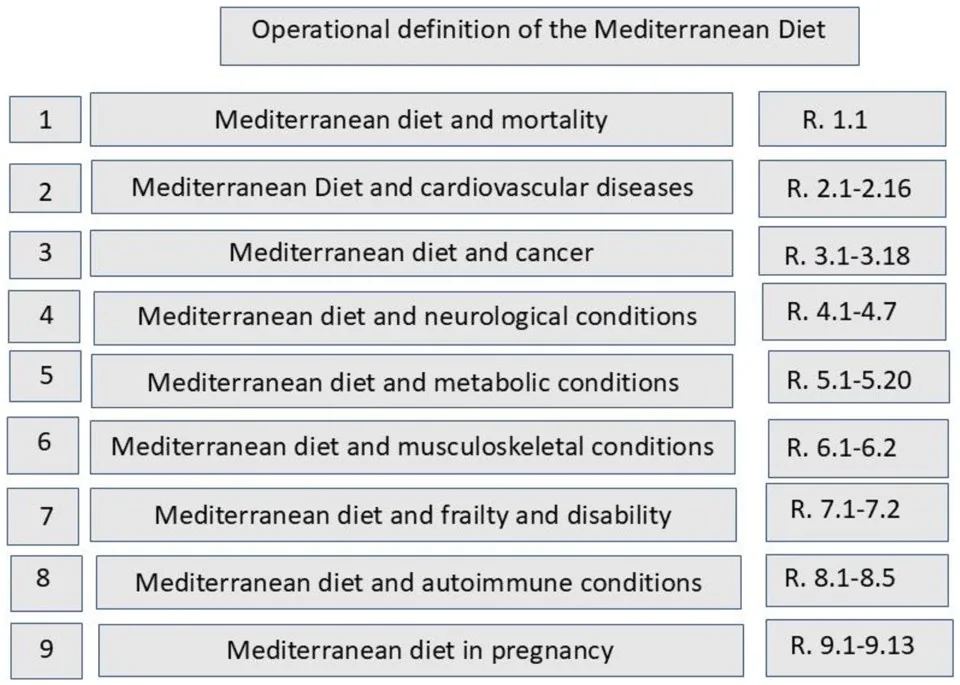
At a Glance: Topics Covered by the Current Guideline

### Study Selection and Data Handling

The analysis included randomized controlled trials (RCTs) in which the MD was the intervention, regardless of the type of control group. In addition, pre/postintervention and observational studies were considered. For observational studies, only those reporting multivariable-adjusted estimates comparing high vs low adherence to the MD were considered. When necessary, effect estimates were standardized to reflect the impact of a 1-point increase in MD adherence, assuming a linear dose–response relationship. When direct estimates were not available, data originally presented (eg, comparisons between higher and lower quantiles) were converted to approximate the effect of a 1-point increase, under the assumption of a linear dose–response relationship.

### Grading and Strength of Recommendations

Each PICO question was evaluated separately for primary and tertiary prevention; secondary prevention was excluded as it pertains to screening practices outside the scope of dietary intervention. The strength of recommendations was assessed using the NUTRIGRADE system, which evaluates the degree of confidence that the benefits of an intervention outweigh potential harms.^11^ Recommendations were classified as either strong (NUTRIGRADE = strong) or weak (NUTRIGRADE = moderate) based on the overall balance of evidence, estimated effect size, and relevant contextual factors. Where evidence strength was rated as low or very low, research recommendations were proposed to guide further investigation. All recommendations were developed through an online multistage consensus process, with agreement percentages (%) recorded. Importantly, the strength of a recommendation was considered along a continuum rather than a binary decision, reflecting the nuanced process of weighing benefits, potential harms, and uncertainties and was not determined solely by the methodological quality of individual studies, but rather by the overall certainty and consistency of the evidence, accounting for study design, methodological rigor, and contribution to the estimated effect size. Strong recommendations were issued when the available evidence consistently demonstrated clear health benefits of the intervention, indicating that most individuals would benefit from its adoption. In these cases, the benefits were judged to clearly outweigh any potential risks or harm. In contrast, weak recommendations were made when there was residual uncertainty about the net benefit, or when the evidence base was insufficient to support a definitive conclusion. In such situations, clinical decisions should be guided by individual patient characteristics, values, and preferences. In accordance with NUTRIGRADE methodology, the strength of each recommendation was determined based on the following criteria: (1) balance of desirable and undesirable outcomes, using the best available estimates of benefit and harm, and considering the clinical relevance of outcomes as perceived by the target population; (2) certainty of the evidence, reflecting the overall methodological quality, consistency, and robustness of the body of evidence; (3) confidence in values and preferences, acknowledging potential variability in how patients prioritize outcomes; (4) resource implications, including the feasibility and cost-effectiveness of implementing the recommendation. This structured framework ensured that the recommendations presented in these guidelines are not only grounded in high-quality evidence but are also aligned with the principles of transparency, clinical relevance, and practical applicability in the fields of nutritional epidemiology and public health.

Recommendation numbers and corresponding NUTRIGRADE ratings are provided in brackets throughout the text. The evidence-to-decision framework tables, which supported the assessment of recommendation strength, are included in Table 1. To further ensure quality and objectivity, the guideline was independently reviewed by 3 external expert referees, whose feedback was carefully considered by the panel members and incorporated into the final version. The development of this guideline was conducted without any external funding or sponsor, ensuring independence and impartiality throughout the process.

**Table 1.** Evidence to Decision Framework

	Q1	Q2	Q3	Q4	Q5	Q6	Q7	Q8	Q9	Q10	Q11	Q12
PICO1	Yes	Wide	Small	Moderate	Probable	Probably intervention	Variable	Probably intervention	Probably greater	Variable	Yes	Yes
PICO2A	Yes	Wide	Small	Low	Possible	Probably intervention	Variable	The intervention	Probably greater	Variable	Yes	Yes
PICO2B	Yes	Wide	Small	Low	Possible	Probably intervention	Variable	The intervention	Probably greater	Variable	Yes	Yes
PICO3A	Yes	Wide	Small	Low	Possible	Probably intervention	Variable	The intervention	Probably greater	Variable	Yes	Yes
PICO3B	Yes	Wide	Small	Low	Possible	Probably intervention	Variable	Don’t know.	Probably greater	Variable	Yes	Yes
PICO4A	Yes	Wide	Small	Low	Possible	Probably intervention	Variable	The intervention	Probably greater	Variable	Yes	Yes
PICO4B	Yes	Wide	Small	Low	Possible	Probably intervention	Variable	Don’t know.	Probably greater	Variable	Yes	Yes
PICO5A	Yes	Wide	Small	Low	Possible	Probably intervention	Variable	I don’t know.	Probably greater	Variable	Yes	Yes
PICO5B	Yes	Wide	Small	Low	Possible	Probably intervention	Variable	The intervention	Probably greater	Variable	Yes	Yes
PICO6A	Yes	Wide	Small	Low	Possible	Probably intervention	Variable	The intervention	Probably greater	Variable	Yes	Yes
PICO6B	Yes	Wide	Small	Moderate	Probable	Probably intervention	Variable	Don’t know.	Probably greater	Variable	Yes	Yes
PICO7A	Yes	Wide	Small	Low	Possible	Probably intervention	Variable	Don’t know.	Probably greater	Yes	Yes	Yes
PICO8A	Yes	Wide	Small	Low	Possible	Probably intervention	Variable	Don’t know.	Probably greater	Variable	Yes	Yes
PICO8B	Yes	Wide	Small	Low	Possible	Probably intervention	Variable	Don’t know.	Probably greater	Variable	Yes	Yes
PICO9	Yes	Wide	Small	Low	Possible	Probably intervention	Variable	Don’t know.	Probably greater	Variable	Yes	Yes

Q1: Is the problem a priority?; Q2: How substantial are the expected desirable effects?; Q3: How substantial are the expected undesirable effects?; Q4: What is the overall certainty of the evidence of effectiveness?; Q5: Is significant uncertainty or variability about the value attributed to the outcome?; Q6: The balance between desirable and undesirable effects favors intervention or the control?; Q7: How large are the resources required?; Q8: Does the cost-effectiveness of intervention favor intervention or control?; Q9: What would be the impact on equity?; Q10: What would be the impact on the environment?; Q11: Is the intervention acceptable to stakeholders?; Q12: Is the implementation of the intervention feasible?.

**Table 2.** PICO 1: What is the Effectiveness of the Mediterranean diet in Reducing Mortality?

No.	Recommendation	Strength	Consensus (%)
Recommendation 1.1	In general population, increased adherence to Mediterranean diet is advisable, as it may contribute to a reduction in all-cause mortality.	Weak	100

**Table 3.** PICO 2A: What is the Effectiveness of the Mediterranean diet for the Primary Prevention of cardiovascular diseases?

#	Recommendation	Strength	Consensus (%)
Recommendation 2.1	For individuals at high cardiovascular risk, MD enriched with EVOO or nuts is recommended over low-fat diet to decrease incidence of peripheral artery disease.	Strong	100
*Recommendation 2.2*	For individuals at high cardiovascular risk, MD enriched with EVOO is recommended over low-fat diet to reduce incidence of atrial fibrillation.	Strong	100
*Recommendation 2.3*	For individuals at high cardiovascular risk, MD enriched with nuts is not recommended over low-fat diet solely to decrease risk of atrial fibrillation.	Strong	100
*Recommendation 2.4*	For individuals at high cardiovascular risk, MD enriched with nuts or with EVOO is not recommended over low-fat diet solely for reducing risk of heart failure.	Strong	100
*Recommendation 2.5*	For individuals at high cardiovascular risk, MD is recommended over low-fat diet to reduce risk of cerebrovascular diseases, such as TIA and ischemic stroke.	Strong	100
*Recommendation 2.6*	For individuals at high cardiovascular risk, MD enriched with nuts is not recommended over low-fat diet solely to decrease risk of coronary heart disease	Strong	100
*Recommendation 2.7*	For individuals at high cardiovascular risk, MD enriched with EVOO or nuts is not recommended over low-fat diet solely to decrease risk of cardiovascular mortality.	Strong	100
*Recommendation 2.8*	In general population, increasing adherence to MD is advisable to help reduce incidence of heart failure.	Weak	100
*Recommendation 2.9*	In general population, increasing adherence to MD is advisable to reduce incidence of hypertension.	Weak	100
*Recommendation 2.10*	In general population, increasing adherence to MD is advisable to decrease incidence of cardiovascular diseases	Weak	100
*Recommendation 2.11*	In general population, increasing adherence to MD is advisable to decrease incidence of coronary heart disease.	Weak	100
*Recommendation 2.12*	In general population, increasing adherence to MD is advisable to decrease incidence of cardiovascular mortality.	Weak	100

Abbreviations: MD, mediterranean diet; EVOO, extra-virgin olive oil.

**Table 4.** PICO 2B: What is the Effectiveness of the Mediterranean diet in People with Cardiovascular Diseases?

#	Recommendation	Strength	**Consensus** **(%)**
*Recommendation 2.13*	In people affected by coronary heart disease, MD is recommended over low-fat diet to decrease cardiovascular mortality.	Strong	100
*Recommendation 2.14*	For individuals with coronary heart disease or heart failure, increasing adherence to MD is advisable to reduce incidence of MACE.	Weak	100
*Recommendation 2.15*	In individuals with coronary heart disease, MD is recommended over low-fat diet to reduce incidence of MACE.	Weak	100
*Recommendation 2.16*	In individuals with cardiovascular disease, increasing adherence to MD is advisable to reduce overall and cause-specific mortality.	Weak	100

Abbreviations: EVOO, extra virgin olive oil; MACE, major cardiovascular event; MD, Mediterranean diet; TIA, transient ischemic attack.

**Table 5.** PICO 3A: What is Effectiveness of the Mediterranean Diet for the Primary Prevention of Cancer Disease?

#	Recommendation	Strength	**Consensus** **(%)**
*Recommendation 3.1*	In general population, greater adherence to MD is advisable to reduce overall incidence of cancer.	Weak	100
*Recommendation 3.2*	In general population, increasing adherence to MD is advisable to reduce incidence of head and neck cancer.	Weak	100
*Recommendation 3.3*	In general population, increasing adherence to MD is advisable to reduce incidence of oral cancer.	Weak	100
*Recommendation 3.4*	In general population, increasing adherence to MD is advisable to reduce incidence of lung cancer.	Weak	100
*Recommendation 3.5*	In general population, increasing adherence to MD is advisable to reduce incidence of gastric cancer.	Weak	100
*Recommendation 3.6*	In general population, increasing adherence to MD is advisable to reduce incidence of liver and gallbladder cancer.	Weak	100
*Recommendation 3.7*	In general population, increasing adherence to MD is advisable to reduce incidence of colorectal cancer.	Weak	100
*Recommendation 3.8*	In general population, increasing adherence to MD is advisable to reduce incidence of bladder cancer.	Weak	100
*Recommendation 3.9*	In general population, increasing adherence to MD is advisable to reduce incidence of breast cancer.	Weak	100
*Recommendation 3.10*	In postmenopausal women, greater adherence to MD enriched with extra-virgin olive oil is recommended over low-fat diet to help reduce risk of breast cancer.	Weak	100
*Recommendation 3.11*	In general population, increasing adherence to MD is advisable to reduce mortality due to cancer.	Weak	100

Abbreviations: MD, Mediterranean diet; MACE, major cardiovascular events;

**Table 6.** PICO 3B: What is Effectiveness of the Mediterranean Diet in People with Oncological Disease?

#	Recommendation	Strength	**Consensus** **(%)**
*Recommendation 3.12*	In patients with colorectal and gynecological cancers, increasing adherence to MD is advisable to reduce all-cause mortality.	Weak	100
*Recommendation 3.13*	In patients with head and neck cancer, increasing adherence to MD is advisable to reduce all-cause mortality.	Weak	100
*Recommendation 3.14*	In patients with ovarian cancer, increasing adherence to MD is advisable to reduce all-cause mortality.	Weak	100
*Recommendation 3.15*	In patients with prostate cancer, increasing adherence to MD is advisable to reduce all-cause mortality.	Weak	100
*Recommendation 3.16*	In patients with breast cancer, it is reasonable to increase adherence to MD to reduce all-cause mortality.	Weak	100
*Recommendation 3.17*	In patients with breast cancer, increasing adherence to MD is advisable to increase disease-free survival.	Weak	100
*Recommendation 3.18*	In patients with gastric cancer, increasing adherence to MD is advisable to reduce all-cause mortality.	Weak	100

**Table 7.** PICO 4A: What is Effectiveness of the Mediterranean Diet for the Primary Prevention of Neurological Disease?

#	Recommendation	Strength	**Consensus** **(%)**
*Recommendation 4.1*	In the general population, greater adherence to MD is advisable to reduce prevalence and incidence of mild cognitive impairment.	Weak	100
*Recommendation 4.2*	In general population, increasing adherence to MD is advisable to reduce incidence of Alzheimer disease.	Weak	100
*Recommendation 4.3*	In general population, increasing adherence to MD is advisable to reduce incidence of dementia.	Weak	100
*Recommendation 4.4*	In general population, increasing adherence to MD is advisable to reduce prevalence of anxiety.	Weak	100
*Recommendation 4.5*	In general population, increasing adherence to MD is advisable to reduce prevalence and incidence of depression.	Weak	100
*Recommendation 4.6*	In general population, increasing adherence to MD is advisable to reduce incidence of Parkinson disease.	Weak	100

**Table 8.** PICO 4B: What is Effectiveness of the Mediterranean Diet in People with Neurological Disease?

#	Recommendation	Strength	Consensus (%)
*Recommendation 4.7*	In patients with Alzheimer disease, increasing adherence to MD is advisable to reduce all-cause mortality.	Weak	100

**Table 9.** PICO 5A: What is Effectiveness of Mediterranean Diet for the Primary Prevention of Metabolic Disease?

#	Recommendation	Strength	**Consensus** **(%)**
*Recommendation 5.1*	In individuals at high cardiovascular risk, MD enriched with EVOO is recommended over low-fat diet to decrease risk of type 2 diabetes mellitus.	Strong	100
*Recommendation 5.2*	In people not affected by diabetes, increasing adherence to MD is advisable to decrease prevalence and incidence of type 2 diabetes mellitus.	Weak	100
*Recommendation 5.3*	In normal-weight people, greater adherence to MD is advisable to reduce prevalence and incidence of overweight and obesity.	Weak	100
*Recommendation 5.4*	In people without metabolic syndrome, increasing adherence to MD is advisable to decrease its incidence.	Weak	100
*Recommendation 5.5*	In normal-weight and overweight people, increasing adherence to MD is advisable to decrease prevalence of hyperuricemia.	Weak	100

Abbreviations: EVOO, extra virgin olive oil; MD, Mediterranean Diet

**Table 10.** PICO 5B: What is Effectiveness of the Mediterranean Diet in People with Metabolic Diseases?

#	Recommendation	Strength	**Consensus** **(%)**
*Recommendation 5.6*	In people with type 2 diabetes or metabolic syndrome, increasing adherence to MD is advisable to decrease risk of all-cause mortality.	Weak	100
*Recommendation 5.7*	In people with NAFLD, a low-calorie MD is recommended over a low-fat diet or standard diet to decrease body mass index.	Weak	100
*Recommendation 5.8*	In people with obesity, MD is recommended over low-fat and standard diet to decrease body mass index.	Weak	100
*Recommendation 5.9*	In people with obesity, MD is recommended over standard diet to decrease serum values of total and LDL cholesterol.	Weak	100
*Recommendation 5.10*	In people with overweight/obesity/diabetes mellitus type 2, MD is recommended over low-fat and standard diet to decrease serum triglyceride levels and to increase serum HDL cholesterol levels.	Weak	100
*Recommendation 5.11*	In children with obesity, MD is recommended over standard diet to decrease serum triglyceride levels.	Weak	100
*Recommendation 5.12*	In children with abdominal obesity, MD is recommended over standard diet to decrease fat mass.	Weak	100
*Recommendation 5.13*	In people with overweight/obesity/diabetes mellitus type 2, MD is recommended over standard diet to decrease fasting serum blood glucose levels.	Weak	100
*Recommendation 5.14*	In people with type 2 diabetes mellitus, MD is recommended over low-fat or standard diabetic diet to achieve greater reductions in HbA1c levels.	Weak	100
*Recommendation 5.15*	In people with type 2 diabetes mellitus, MD is recommended over low-fat or standard diet to decrease HOMA-IR values, indicating improved insulin sensitivity	Weak	100
*Recommendation 5.16*	In people with NAFLD, MD is recommended over low-fat or standard diet to decrease waist circumference.	Weak	100
*Recommendation 5.17*	In people with prediabetes, MD is recommended over standard diet to decrease body weight.	Weak	100
*Recommendation 5.18*	In people with type 2 diabetes, MD is recommended over low-fat or standard diet to decrease serum C-reactive protein levels.	Weak	100
*Recommendation 5.19*	In people with NAFLD, MD is recommended over low-fat diet to decrease serum C-reactive protein levels.	Weak	100
*Recommendation 5.20*	In people with NAFLD, MD is recommended over low-fat diet to improve health related quality of life.	Weak	100

**Table 11.** PICO 6A: What is Effectiveness of the Mediterranean Diet for the Primary Prevention of Musculoskeletal Diseases?

#	Recommendation	Strength	Consensus (%)
*Recommendation 6.1*	In general population, increasing adherence to MD is advisable to reduce incidence of fractures.	Weak	100

**Table 12.** PICO 6B: What is Effectiveness of the Mediterranean Diet in People with Musculoskeletal Disease?

#	Recommendation	Strength	Consensus (%)
*Recommendation 6.2*	In people with osteoarthritis, increasing adherence to MD is advisable to reduce pain.	Weak	100

**Table 13.** PICO 7A: What is Effectiveness of the Mediterranean Diet for the Primary Prevention of Disability and Frailty in Older People?

#	Recommendation	Strength	Consensus (%)
*Recommendation 7.1*	In older individuals, increasing adherence to MD is advisable to reduce incidence of frailty.	Weak	100
*Recommendation 7.2*	In older individuals, increasing adherence to MD is advisable to reduce prevalence of disability.	Weak	100

**Table 14.** PICO 8A: What is Effectiveness of the Mediterranean Diet for the Primary Prevention of Autoimmune Disease?

#	Recommendation	Strength	Consensus (%)
*Recommendation 8.1*	In general population, greater adherence to MD is advisable to reduce incidence of multiple sclerosis.	Weak	100

**Table 15.** PICO 8B: What is Effectiveness of the Mediterranean Diet in People with Autoimmune Disease?

#	Recommendation	Strength	Consensus (%)
*Recommendation 8.2*	In adults with Crohn disease, increasing adherence to MD is advisable to reduce all-cause mortality.	Weak	100
*Recommendation 8.3*	In adults with multiple sclerosis, increasing adherence to MD is advisable to increase health related quality of life.	Weak	100
*Recommendation 8.4*	In adolescents and adults with celiac disease, increasing adherence to MD is advisable to increase health related quality of life.	Weak	100
*Recommendation 8.5*	In adults with ulcerative colitis, increasing adherence to MD is advisable to increase health related quality of life.	Weak	100

**Table 16.** PICO 9: What is Effectiveness of the Mediterranean Diet in Pregnant Women?

#	Recommendation	Strength	Consensus (%)
*Recommendation 9.1*	In pregnant women, MD is recommended over low-fat or standard diet to decrease risk of small for gestational age newborns.	Strong	100
*Recommendation 9.2*	In pregnant women, MD is recommended over low-fat diet to decrease risk of large for gestational age newborns.	Strong	100
*Recommendation 9.3*	In pregnant women, MD is recommended over low-fat or standard diet to decrease risk of gestational diabetes.	Strong	100
*Recommendation 9.4*	In pregnant women, increasing adherence to MD is advisable to decrease risk of fetal growth disorders.	Weak	100
*Recommendation 9.5*	In pregnant women, greater adherence to MD is advisable to decrease risk of small for gestational age	Weak	100
*Recommendation 9.6*	In pregnant women, increasing adherence to MD is advisable to decrease risk of large for gestational age	Weak	100
*Recommendation 9.7*	In pregnant women, increasing adherence to MD is advisable to decrease risk of gestational diabetes.	Weak	100
*Recommendation 9.8*	In pregnant women, we do not recommend MD over low-fat and standard diets solely to reduce risk of respiratory distress at birth.	Weak	100
*Recommendation 9.9*	In pregnant women, MD is recommended over low-fat or standard diet to decrease risk of maternal infections.	Weak	100
*Recommendation 9.10*	In pregnant women, we do not recommend MD over standard diet solely to decrease risk of newborn death.	Weak	100
*Recommendation 9.11*	In pregnant women, MD is recommended over low-fat or standard diets to decrease risk of preterm delivery.	Weak	100
*Recommendation 9.12*	In pregnant women, increasing adherence to MD is advisable to reduce risk of preterm delivery.	Weak	100
*Recommendation 9.13*	In pregnant women, we do not recommend MD over low-fat diet sole purpose of decreasing risk of pre-eclampsia.	Weak	100
*Recommendation 9.14*	In pregnant women, increasing adherence to MD is advisable to reduce risk of pre-eclampsia.	Weak	100

#### Organization of the Writing Committee

The development of these guidelines was overseen by a multidisciplinary writing committee composed of experts in clinical medicine, epidemiology, nutrition, geriatrics, public health, and health economics. The committee included representatives from over 20 Italian scientific societies, as well as members from national health institutions and stakeholder organizations. The committee was co-chaired by leading figures in cardiovascular prevention, clinical nutrition, and public health, ensuring both scientific rigor and applicability to real-world settings. Committee members were selected based on their subject-matter expertise, methodological competence, and experience in guideline development. To guarantee a comprehensive and balanced perspective, the writing group also included professionals with experience in primary care, hospital-based medicine, and preventive services, as well as academics and researchers involved in dietary and lifestyle-related interventions. Each member of the writing committee was assigned to 1 or more PICO topic areas, for which they served as primary authors or reviewers, depending on their domain expertise. Assignments were made to ensure that each PICO area benefited from multidisciplinary insights and high-level scientific oversight. To support methodological transparency, all committee members disclosed potential conflicts of interest, which were reviewed and approved in accordance with the policies of the Istituto Superiore di Sanità.

The committee’s work was supported by an independent methodological board, which coordinated the systematic evidence review, conducted quality grading using the NUTRIGRADE tool, and managed the application of the Evidence-to-Decision framework. The process also included contributions from a health economics team, which provided input on resource use and sustainability issues related to MD implementation. An external panel of reviewers, composed of internationally recognized experts in nutrition, chronic disease prevention, and evidence-based medicine, conducted an independent appraisal of the guideline and submitted formal comments, which were incorporated into the final version of the document. This collaborative and inclusive approach ensured that the recommendations are not only grounded in scientific evidence, but also reflect the values, priorities, and practical realities of multiple professional and public health contexts.

### Document Review and Approval

This guideline underwent a structured, transparent peer-review and approval process in accordance with international best practices for developing clinical recommendations. A draft was submitted for external evaluation by a panel of 3 independent expert referees, selected for their recognized expertise in nutrition, epidemiology, and chronic disease prevention, as well as their clinical and methodological competence. These reviewers were nominated by the promoting organizations, and each completed a detailed conflict of interest disclosure, assessed in accordance with the ISS standards for transparency and integrity. Feedback from the external reviewers was systematically reviewed and discussed by the writing committee. Substantive revisions were made to enhance the document’s scientific rigor, clarity, and practical relevance of the final recommendations.

The final guideline was formally approved by the governing bodies of the promoting societies and by endorsing stakeholders. No commercial or industry funding was involved at any stage of the development process. This independent, multi-tiered review ensures that these recommendations are scientifically robust, aligned with public health priorities, and reflect a consensus among Italy’s leading experts and institutions in nutrition and chronic disease prevention.

## RECOMMENDATIONS

The following sections present topic-specific recommendations derived from the guideline. From a methodological point of view, the recommendations, based on the revision of the literature, were discussed in the light of the actual Italian National Health System. Detailed analyses supporting these recommendations will be addressed in forthcoming systematic reviews and meta-analyses developed in alignment with this work.

### Operational Definition of MD

#### Grade of Recommendation GPP—Strong Consensus (100% Agreement)

The MD is characterized by a predominantly plant-based dietary pattern, emphasizing high consumption vegetables, fresh fruits, nuts, legumes, nuts, and whole grains. Extra virgin olive oil (EVOO), rich in monounsaturated fatty acids and phytochemicals, serves as the primary source of dietary fat. The diet also includes moderate consumption of fish, seafood, eggs, white meat, and milk and other dairy products, while intake of red and processed meat and sweets is limited. Traditionally, the MD dietary pattern reflects food choices rooted in the consumption of seasonal, locally sourced, and minimally processed ingredients. However, the MD extends beyond dietary components to encompass a broader lifestyle that includes moderate, regular physical activity; adequate rest; and conviviality and mindful eating practices. The MD aligns with principles of biodiversity, environmental sustainability, and support for local food systems. Furthermore, the MD is recognized as a cultural model that fosters social cohesion and intergenerational connections through shared meals and communal food traditions. It should be acknowledged that the MD does not represent a single, homogeneous dietary pattern. Instead, its practical expression differs across Mediterranean countries, depending on local biodiversity, culinary traditions, and cultural habits. For example, the relative predominance of cereals, legumes, or specific vegetables varies between Northern Africa, the Middle East, and Southern Europe. Despite these regional differences, the MD is unified by shared principles—such as the central role of EVOO, the emphasis on plant-based and minimally processed foods, and the cultural practices of conviviality and mindful eating—that collectively define its health-promoting and sustainable nature. In this sense, the MD also shares affinities with other traditional dietary models that integrate food, culture, and social practices. This integrative and sustainable approach contributes to the established role of the MD in promoting health and well-being and underpins its designation as an intangible cultural heritage by UNESCO.

One of the most consistent and compelling findings is the significant association between greater adherence to the MD and a reduced risk of all-cause mortality in the general population. This protective effect is plausibly mediated by the MD’s influence on key biological pathways, including the regulation of lipid and glucose metabolism, the reduction of systemic inflammation, and the mitigation of oxidative stress, all of which are critical factors in the development and progression of multiple NCDs and premature mortality.^12^

The MD plays a crucial role in reducing the incidence of various cardiovascular conditions, including peripheral artery disease (PAD), atrial fibrillation (AF), and stroke/transient ischemic attack (TIA), probably through its lipid-lowering, anti-inflammatory, and vascular-protective effects.^13^ For instance, a sub-analysis of the PREDIMED trial found that participants following an MD enriched with nuts experienced a 50% lower risk of developing PAD compared to those on a low-fat diet.^14^ In the context of AF, the diet’s emphasis on omega-3 fatty acids from fish, antioxidants from fruits and vegetables, and avoidance of processed foods may reduce oxidative stress and atrial remodeling—2 factors involved in AF development.^15^ Supporting this finding, a 2014 analysis of the PREDIMED cohort found that greater adherence to the MD was significantly associated with a reduced risk of AF in older adults.^16^ Additionally, the MD has been consistently linked to a lower risk of stroke. Its positive effects on blood pressure, glycemic control, and endothelial function contribute to preventing ischemic stroke, which is often caused by atherosclerosis and embolic events.^17^ Finally, moderate-quality evidence from primary prevention studies suggest that the MD modestly reduces the risk of hypertension, heart failure, and coronary heart disease in the broader population.

In tertiary prevention, ie, in people already affected by CVD, higher adherence to the MD modestly improves some cardiovascular outcomes but strongly reduces mortality among individuals with established heart disease, as has been shown in multiple RCTs.^18^^,^^19^

The primary prevention of cancer is a public health priority. Unhealthy dietary patterns are strongly associated with a higher risk of certain cancers, particularly those affecting the gastrointestinal tract.^20^ Current evidence supports the protective role of the MD against a spectrum of malignancies, including colorectal, breast, head and neck, gastric, liver, lung, and oral cancers. While much of this evidence is of moderate quality, the consistency of findings across various cancer types highlights the broad-spectrum anti-carcinogenic potential of the MD, likely mediated by its insulin sensitizing, anti-inflammatory, antioxidant, and fiber-rich properties.^21^^,^^22^

In cancer survivors, higher adherence to the MD correlates with reduced all-cause mortality and improved disease-free survival, particularly in breast and colorectal cancer, emphasizing the growing role of dietary interventions in oncology care.

The MD also demonstrates neuroprotective effects, with consistent evidence of reduced risk for mild cognitive impairment, Alzheimer disease, various forms of dementia, Parkinson disease, depression, and anxiety. Beyond the mechanisms shared with cardiovascular prevention, these associations are biologically plausible due to the richness of the MD in vitamins, potassium, magnesium, omega-3 fatty acids, and polyphenols, which have been shown to lower systolic blood pressure and mitigate neuroinflammation and oxidative stress.^23^^,^^24^ Furthermore, in the context of tertiary prevention, higher adherence to the MD in individuals with Alzheimer disease has been associated with lower all-cause mortality, although further interventional studies are warranted to establish causality.

Metabolic diseases include a range of conditions such as obesity, metabolic syndrome, type 2 diabetes and dyslipidemia. In individuals at high cardiovascular risk, an MD enriched with EVOO has demonstrated superior benefits over a traditional low-fat diet in reducing the incidence of type 2 diabetes mellitus.^25^ Specifically, individuals randomized to the intervention group showed a 40% lower risk of diabetes onset, even without intentional weight loss or increased physical activity. This protective effect is largely attributed to the high content of monounsaturated fats in EVOO, which improves insulin sensitivity, as well as the abundance of antioxidant and anti-inflammatory compounds that help regulate glucose metabolism.^25^ These findings strongly support the recommendation of a MD rich in EVOO for individuals at elevated cardiovascular risk, not only for cardiac health but also for effective type 2 diabetes prevention.

In individuals with established T2DM and obesity, the MD is effective in improving glycemic control (ie, reductions in HbA1c, HOMA-IR), lipid profile (ie, decreased LDL cholesterol and triglycerides), and anthropometric measures (eg, BMI and waist circumference)^22^, especially when combined with calorie restriction as shown in the PREDIMED-Plus trial.^26^ Importantly, these benefits extend to children and adolescents, including reductions in triglyceride levels in children with obesity and improvements in metabolic outcomes in those with non-alcoholic fatty liver disease.^27^ Collectively, these findings affirm the MD as a foundational component of dietary therapy for metabolic syndrome, prediabetes/diabetes, insulin resistance and related disorders.

These guideline underscores the beneficial role of the MD in musculoskeletal health, as reflected in Recommendations 6.1 and 6.2, which support its effectiveness in reducing fracture risk in general population and alleviating osteoarthritis symptoms, such as pain. These benefits are likely mediated by the diet’s anti-inflammatory properties and its abundance in bone-supportive nutrients such as calcium, magnesium, and vitamin K—factors that align with findings from observational studies and small-scale clinical trials.^28^

In older adults, MD adherence is associated with lower risk of frailty and disability (Recommendations 7.1 and 7.2), likely mediated through preservation of muscle mass, better micronutrient intake, and systemic anti-inflammatory effects.^29^

Emerging moderate-quality evidence suggests a protective role for the MD in autoimmune diseases. Among the general population, increased MD adherence is associated with a reduced risk of multiple sclerosis (Recommendation 8.1). Furthermore, among individuals with Crohn’s disease, ulcerative colitis, multiple sclerosis, and celiac disease, MD adherence is linked to improved quality of life and reduced mortality (Recommendations 8.2–8.5). These benefits may be attributed to the MD’s capacity to modulate gut microbiota composition and immune function—key elements in the pathophysiology of autoimmune diseases. The MD’s high fiber content promotes the production of short-chain fatty acids, which play a crucial role in maintaining intestinal barrier integrity, reducing inflammation, and supporting immune regulation.^30^

During pregnancy, adherence to the MD significantly decreases the risk of fetal growth abnormalities, gestational diabetes, maternal infections, preterm birth, and small-for-gestational-age infants. Notably, 3 recommendations are supported by high-level evidence from randomized controlled trials, endorsing the MD over other dietary patterns for the prevention of onset of SGA (small-for-age) or LGA (large-for-age) newborns or diabetes. These findings underscore the critical role of balanced macronutrient intake, reduced glycemic load, and improved micronutrient status in optimizing maternal and neonatal outcomes.

## CONCLUSIONS

This guideline reaffirms the MD as a cornerstone of lifelong health promotion. Its benefits span all life stages and a broad range of disease processes, supporting both the prevention and management of cardiovascular, metabolic, oncologic, neurodegenerative, musculoskeletal, autoimmune, and perinatal conditions. Of importance, we found that in patients at high risk of CVD, such as those included in the PREDIMED study, the MD particularly when supplemented with EVOO or nuts, significantly reduces the incidence of PAD, AF, stroke/TIA and type 2 diabetes (Figure 2**)**. Furthermore, it has demonstrated a protective role against abnormal birth weight in newborns and type 2 diabetes, as summarized in Figure 3.

**Figure 2.**
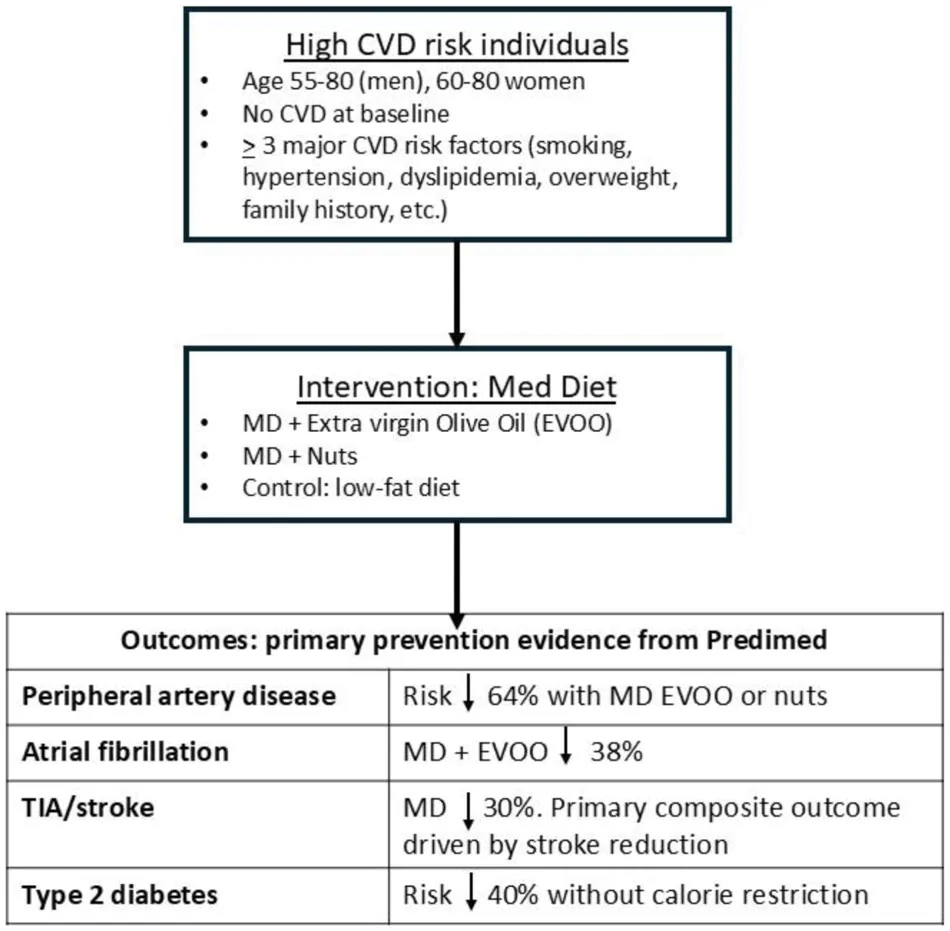
Evidence of Mediterranean Diet in Individuals at High Risk of Cardiovascular Disease

**Figure 3.**
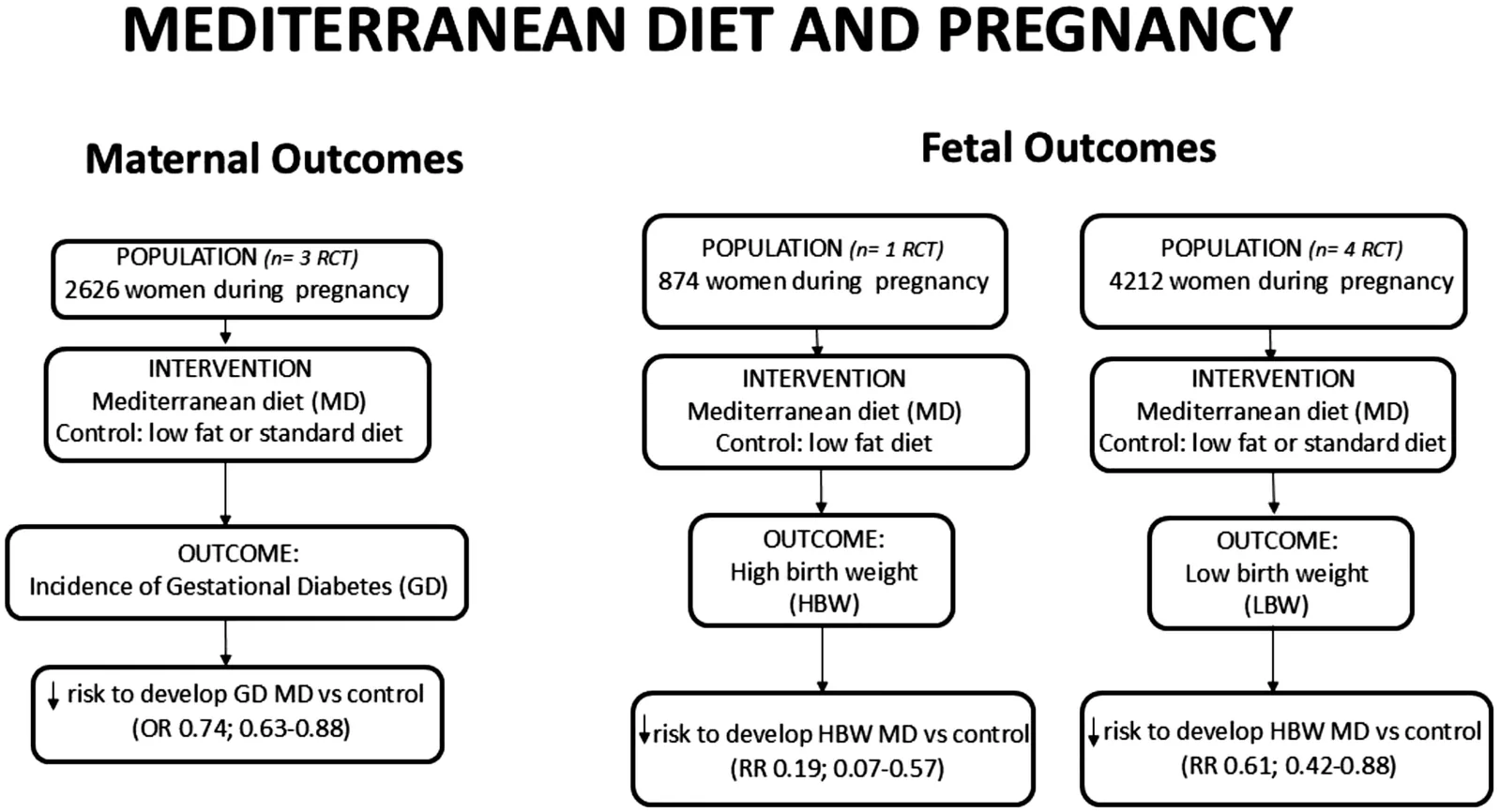
Evidence of Mediterranean Diet in Pregnant Women

These findings align closely with the World Health Organization framework for healthy aging, highlighting the importance of integrating nutritional strategies into broader public health strategies, alongside smoking cessation, physical activity promotion, and vaccination programs.^31^ With 84 evidence-based clinical recommendations, this guideline represents what is to our knowledge one of the most comprehensive and scientifically grounded evaluations of the MD to date. It offers a practical, actionable and evidence-informed roadmap for clinicians, policymakers, and the public, positioning the MD as one of the most effective and well-supported dietary patterns available. By bridging the gap between prevention and therapy, this work establishes a strong foundation for the widespread adoption of the MD as a central component of global health strategies.

## References

[1] Dominguez LJ , Veronese N, Di Bella G, et al Mediterranean diet in the management and prevention of obesity. Exp Gerontol. 2023;174:112121. 10.1016/j.exger.2023.112121 36792040

[2] Dominguez L , Galioto A, Ferlisi A, et al Ageing, lifestyle modifications, and cardiovascular disease in developing countries. J Nutr Health Aging. 2006;10:143. 16554951

[3] Fontana L , Fasano A, Chong YS, Vineis P, Willett WC. Transdisciplinary research and clinical priorities for better health. PLoS Med. 2021;18:e1003699. 10.1371/journal.pmed.1003699 PMC8315511 34314418

[4] Cheng K , Fontana L. Investing in value-based primary care: a pathway to sustainable healthcare. Eur Heart J. 2024;45:3103-3105. 10.1093/eurheartj/ehae404 38984386

[5] Jacobs DR Jr. , Gross MD, Tapsell LC. Food synergy: an operational concept for understanding nutrition. Am J Clin Nutr. 2009;89:1543S-1548S. 10.3945/ajcn.2009.26736B PMC2731586 19279083

[6] Hu FB , Drescher G, Trichopoulou A, Willett WC, Martínez-González MA. Three decades of the mediterranean diet pyramid: a narrative review of its history, evolution, and advances. Am J Clin Nutr. 2025;122:17-28. 10.1016/j.ajcnut.2025.04.036 PMC13639439 40374164

[7] Trichopoulou A , Martínez-González MA, Tong TY, et al Definitions and potential health benefits of the Mediterranean diet: views from experts around the world. BMC Med. 2014;12:112-116. 10.1186/1741-7015-12-112 PMC4222885 25055810

[8] Dernini S , Berry EM. Mediterranean diet: from a healthy diet to a sustainable dietary pattern. Front Nutr. 2015;2:15. 10.3389/fnut.2015.00015 PMC4518218 26284249

[9] Cardamone E , Iacoponi F, Di Benedetto R, et al Adherence to Mediterranean Diet and its main determinants in a sample of Italian adults: results from the ARIANNA cross-sectional survey. Front Nutr. 2024;11:1346455. 10.3389/fnut.2024.1346455 PMC10927747 38476598

[10] Page MJ , McKenzie JE, Bossuyt PM, et al The PRISMA 2020 statement: an updated guideline for reporting systematic reviews. BMJ. 2021;372:n71. 10.1136/bmj.n71 PMC8005924 33782057

[11] Schwingshackl L , Knüppel S, Schwedhelm C, et al Perspective: NutriGrade: A scoring system to assess and judge the meta-evidence of randomized controlled trials and cohort studies in nutrition research. Adv Nutr. 2016;7:994-1004. 10.3945/an.116.013052 PMC5105044 28140319

[12] Górska P , Górna I, Przysławski J. Mediterranean diet and oxidative stress. NFS. 2021;51:677-689.

[13] Tosti V , Bertozzi B, Fontana L. Health benefits of the Mediterranean diet: metabolic and molecular mechanisms. J Gerontol A Biol Sci Med Sci. 2018;73:318-326. 10.1093/gerona/glx227 PMC7190876 29244059

[14] Ruiz-Canela M , Estruch R, Corella D, Salas-Salvadó J, Martínez-González MA. Association of Mediterranean diet with peripheral artery disease: the PREDIMED randomized trial. JAMA. 2014;311:415-417. 10.1001/jama.2013.280618 24449321

[15] Ahmed A , Arshad MS, Khan MS. Trimming the fat, soothing the heart: mediterranean diet as a potential game-changer for prevention of atrial fibrillation?. Eur J Prev Cardiol. 2024;31:346-347. 10.1093/eurjpc/zwad379 38064582

[16] Martínez-González M , Toledo E, Arós F, et al; PREDIMED Investigators. Extravirgin olive oil consumption reduces risk of atrial fibrillation: the PREDIMED (Prevención con Dieta Mediterránea) trial. Circulation. 2014;130:18-26. 10.1161/circulationaha.113.006921 24787471

[17] Estruch R , Ros E, Salas-Salvadó J, et al; PREDIMED Study Investigators. Primary prevention of cardiovascular disease with a mediterranean diet supplemented with extra-virgin olive oil or nuts. N Engl J Med. 2018;378:e34. 10.1056/NEJMoa1800389 29897866

[18] de Lorgeril M , Salen P, Martin JL, Monjaud I, Delaye J, Mamelle N. Mediterranean diet, traditional risk factors, and the rate of cardiovascular complications after myocardial infarction: final report of the Lyon Diet Heart Study. Circulation. 1999;99:779-785. 10.1161/01.cir.99.6.779 9989963

[19] Singh RB , Dubnov G, Niaz MA, et al Effect of an Indo-Mediterranean diet on progression of coronary artery disease in high risk patients (Indo-Mediterranean Diet Heart Study): a randomised single-blind trial. Lancet. 2002;360:1455-1461. 10.1016/s0140-6736(02)11472-3 12433513

[20] Grosso G , Bella F, Godos J, et al Possible role of diet in cancer: systematic review and multiple meta-analyses of dietary patterns, lifestyle factors, and cancer risk. Nutr Rev. 2017;75:405-419. 10.1093/nutrit/nux012 28969358

[21] Giordano G , Mastrantoni L, Terranova R, Colloca GF, Zuccalà G, Landi F. The role of Mediterranean diet in cancer incidence and mortality in the older adults. NPJ Aging. 2024;10:61-11. 10.1038/s41514-024-00186-w PMC11621705 39639020

[22] Tosti V , Bertozzi B, Fontana L. Health benefits of the Mediterranean diet: metabolic and molecular mechanisms. J Gerontol: Series A. 2017;73:318-326. 10.1093/gerona/glx227 PMC7190876 29244059

[23] Picone P , Girgenti A, Buttacavoli M, Nuzzo D. Enriching the Mediterranean diet could nourish the brain more effectively. Front Nutr. 2024;11:1489489. 10.3389/fnut.2024.1489489 PMC11631615 39664911

[24] Nucci D , Sommariva A, Degoni LM, et al Association between Mediterranean diet and dementia and Alzheimer disease: a systematic review with meta-analysis. Aging Clin Exp Res. 2024;36:77. 10.1007/s40520-024-02718-6 PMC10959819 38519775

[25] Salas-Salvadó J , Bulló M, Estruch R, et al Prevention of diabetes with Mediterranean diets: a subgroup analysis of a randomized trial. Ann Intern Med. 2014;160:1-10. 10.7326/M13-1725 24573661

[26] Martínez-Montoro JI , Cornejo-Pareja I, Díaz-López A, et al; PREDIMED‐Plus Investigators. Effect of an intensive lifestyle intervention on cystatin C-based kidney function in adults with overweight and obesity: from the PREDIMED-Plus trial. J Intern Med. 2025;297:141-155. 10.1111/joim.20038 PMC11771574 39723807

[27] Katsagoni CN , Papachristou E, Sidossis A, Sidossis L. Effects of dietary and lifestyle interventions on liver, clinical and metabolic parameters in children and adolescents with non-alcoholic fatty liver disease: a systematic review. Nutrients. 2020;12:2864. 10.3390/nu12092864 PMC7551480 32961669

[28] Veronese N , Ragusa FS, Dominguez LJ, Cusumano C, Barbagallo M. Mediterranean diet and osteoarthritis: an update. Aging Clin Exp Res. 2024;36:231-210. 10.1007/s40520-024-02883-8 PMC11614952 39625615

[29] Andreo-López MC , Contreras-Bolívar V, García-Fontana B, García-Fontana C, Muñoz-Torres M. The influence of the mediterranean dietary pattern on osteoporosis and sarcopenia. Nutrients. 2023;15:3224. 10.3390/nu15143224 PMC10385532 37513646

[30] Xie L , Alam MJ, Marques FZ, Mackay CR. A major mechanism for immunomodulation: dietary fibres and acid metabolites. Semin Immunol. 2023;66:101737. 10.1016/j.smim.2023.101737 36857894

[31] Beard JR , Officer A, De Carvalho IA, et al The World report on ageing and health: a policy framework for healthy ageing. Lancet. 2016;387:2145-2154. 10.1016/S0140-6736(15)00516-4 PMC4848186 26520231

